# Exogenous Expression of Human apoA-I Enhances Cardiac Differentiation of Pluripotent Stem Cells

**DOI:** 10.1371/journal.pone.0019787

**Published:** 2011-05-11

**Authors:** Kwong-Man Ng, Yee-Ki Lee, Wing-Hon Lai, Yau-Chi Chan, Man-Lung Fung, Hung-Fat Tse, Chung-Wah Siu

**Affiliations:** 1 Stem Cell & Regenerative Medicine Program, Research Centre of Heart, Brain, Hormone and Healthy Ageing, Li Ka Shing Faculty of Medicine, University of Hong Kong, Hong Kong; 2 Department of Physiology, University of Hong Kong, Hong Kong; 3 Cardiology Division, Department of Medicine, Queen Mary Hospital, University of Hong Kong, Hong Kong; Baylor College of Medicine, United States of America

## Abstract

The cardioprotective effects of high-density lipoprotein cholesterol (HDL-C) and apolipoprotein A1 (apoA-I) are well documented, but their effects in the direction of the cardiac differentiation of embryonic stem cells are unknown. We evaluated the effects of exogenous apoA-I expression on cardiac differentiation of ESCs and maturation of ESC-derived cardiomyocytes. We stably over-expressed full-length human apoA-I cDNA with lentivirus (LV)-mediated gene transfer in undifferentiated mouse ESCs and human induced pluripotent stem cells. Upon cardiac differentiation, we observed a significantly higher percentage of beating embryoid bodies, an increased number of cardiomyocytes as determined by flow cytometry, and expression of cardiac markers including α-myosin heavy chain, β-myosin heavy chain and myosin light chain 2 ventricular transcripts in LV-apoA-I transduced ESCs compared with control (LV-GFP). In the presence of noggin, a BMP4 antagonist, activation of BMP4-SMAD signaling cascade in apoA-I transduced ESCs completely abolished the apoA-I stimulated cardiac differentiation. Furthermore, co-application of recombinant apoA-I and BMP4 synergistically increased the percentage of beating EBs derived from untransduced D3 ESCs. These together suggests that that pro-cardiogenic apoA-I is mediated via the BMP4-SMAD signaling pathway. Functionally, cardiomyocytes derived from the apoA-I-transduced cells exhibited improved calcium handling properties in both non-caffeine and caffeine-induced calcium transient, suggesting that apoA-I plays a role in enhancing cardiac maturation. This increased cardiac differentiation and maturation has also been observed in human iPSCs, providing further evidence of the beneficial effects of apoA-I in promoting cardiac differentiation. In Conclusion, we present novel experimental evidence that apoA-I enhances cardiac differentiation of ESCs and iPSCs and promotes maturation of the calcium handling property of ESC-derived cardiomyocytes via the BMP4/SMAD signaling pathway.

## Introduction

Pluripotent stem cells, including embryonic stem cells (ESCs) and induced pluripotent stem cells (iPSCs), can differentiate into virtually all cells of the body including cardiomyocytes. They thus represent an unlimited *ex-vivo* cell source for cardiac regenerative therapy [1]. Spontaneous differentiation of pluripotent stem cells toward cardiac lineage is nonetheless inefficient, and the resultant cardiomyocytes display immature electrophysiological and calcium handling properties [2], [3], [4]. These immaturities result in ineffective contractile force generation and may also create arrhythmogenic substrates. This raises potential safety concerns for cell-based cardiac therapy [1], [5].

High-density lipoprotein-cholesterol (HDL-C) and its principle component, apolipoprotein A-1 (apoA-I), have been consistently demonstrated by large prospective epidemiological studies to be two of the most powerful independent negative predictors of cardiovascular events [6], [7]. With each 1 mg/dL increment of HDL-C, there is a 2 to 3% reduction in risk of coronary artery disease [6], [7]. The beneficial effects of HDL-C/apoA-I are mainly attributed to its role in reversal of cholesterol transport and in regulation of endothelial cell proliferation and migration [8], [9]. This in turn reduces atherosclerotic progression. Recent evidence indicates that HDL-C/apoA-I exerts direct protective effects on adult cardiomyocytes under various stresses through signal pathways that are unrelated to cholesterol transportation [10], [11], [12]. It has been previously reported that fetal cardiomyocytes as well as their adult counterparts express apoA-I, although the physiological significance remains largely unknown [13], [14]. We evaluated the hypothesis that human apoA-I gene-transfer may enhance cardiac differentiation of pluripotent stem cells (murine ESCs and human iPSCs) and facilitate the maturation of calcium handling properties of their cardiac derivatives. Our data indicate a direct cardiogenic effect of apoA-I gene-transfer on mouse ESCs and human iPSCs via a BMP4/SMAD signaling pathway.

## Results

### Exogenous apoA-I expression promotes cardiac differentiation of mouse ESCs

**Figure 1:**
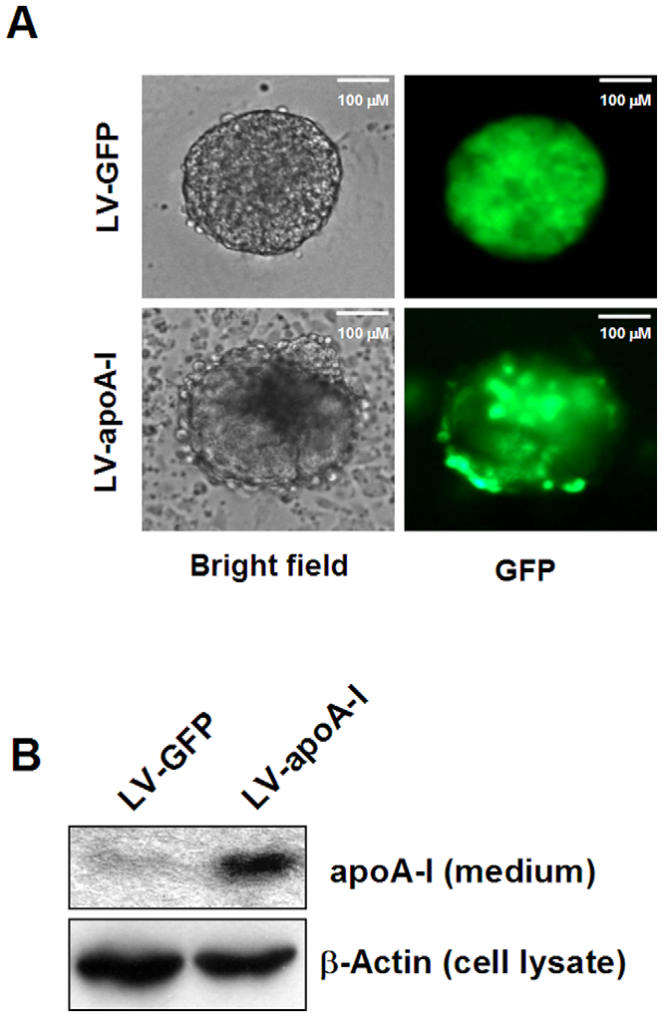
Expression of human apoA-I in D3 mouse ESCs. Undifferentiated ESCs were cultured in the absence of a feeder layer and transduced with lentiviral particles containing the full-length wild type apoA-I cDNA under the control of CMV promoter. This expression cassette was linked to the IRES-GFP reported cassette for the identification of transduced cells, while the lentiviral particles containing the empty construct served as a control. To evaluate the success of gene transfer, undifferentiated empty construct- and apoA-I-transduced cells were examined for expression of GFP (A). The secretion of apoA-I in the concentrated spent medium was determined by Western blot analysis using an antibody specific to apoA-I.

To evaluate the effects of apoA-I on cardiac differentiation of ESCs, we first constructed pHRST-CMV-apoA-I-IRES-GFP (pLV-apoA-I) that allowed simultaneous expression of full-length cDNA of human apoA-I and GFP under the control of CMV promoter. We confirmed the efficacy of our LV-apoA-I construct to mediate exogenous expression of apoA-I in undifferentiated ESCs. Figure 1A shows that transduction of undifferentiated ESCs after lentivirus-mediated gene transfer, (1) LV-apoA-I, and (2) LV-GFP, enabled generation of ESC lines that stably expressed GFP. In addition, apoA-I protein (∼30 kDa) was detected in the medium of LV-apoA-I transduced undifferentiated ESCs but not the LV-GFP counterpart (Figure 1B).

**Figure 2:**
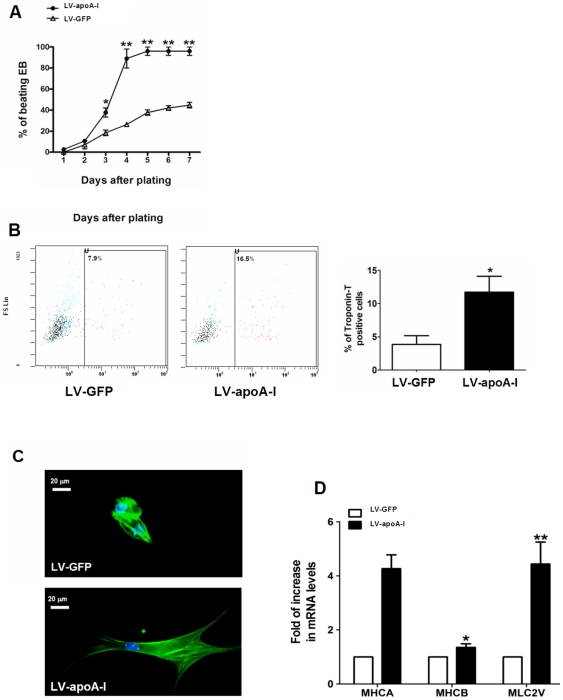
Effect of apoA-I gene transfer on generation of beating embryoid body and cardiac cells. (A) Empty construct and apoA-I-transduced mouse ESCs were differentiated using the conventional “hanging-drop” method, and the resultant embryoid bodies (EBs) were plated onto gelatin coated plates. The occurrence of beating areas within the EBs was observed and counted for 8 days starting from the day of plating. (B) Percentage of ESC-derived cardiomyocytes (troponin-T positive cells) on day 8 as determined by flow cytometry. (C) Individual cardiomyocytes were isolated from the beating area of the EBs and identified with immunnohistochemistry using antibody specific to the cardiac troponin-T. (D) Cardiac maker gene expression in the embryoid bodies derived from empty construct- and apoA-I-transduced ESCs as revealed by real-time quantitative PCR analysis. MHCA: α-myosin heavy chain; MHCB: β-myosin heavy chain; MLC2V: myosin light chain 2 ventricular transcripts. Data shown as mean ± SEM from at least 3 independent experiments, n = 3–5, *p<0.05; **p<0.005.

After confirming the effectiveness of gene transfer, *in vitro* cardiac differentiation was assessed and confirmed when spontaneous beating embryoid bodies were visualized under microscopic and flow cytometry analysis. Although spontaneously beating outgrowths were observed in both LV-GFP- and LV-apoA-I -transduced ESCs as early as day 1, LV- apoA-I-transduced ESCs had a persistently and significantly higher percentage of embryoid bodies containing spontaneously beating outgrowths than LV-GFP-transduced ESCs from day 3 to day 7 (96±4% vs. 45±3%, *p*<0.005)(Figure 2A). Although a commonly used method, counting of beating outgrowths from embryoid bodies is nonetheless a very crude means of assessing cardiac differentiation [15]. To further confirm that LV-apoA-I enhanced cardiac differentiation, flow cytometry to determine the percentage of cardiomyocytes as identified by troponin-T positive cells was performed on day 8. The percentage of cardiomyocytes was consistently and significantly higher in LV-apoA-I transduced ESCs than in LV-GFP transduced cells (12±2% *vs.* 4±1%; *p*<0.05) (Figure 2B). Furthermore, the morphology of LV-GFP-transduced ESC-derived cardiomyocytes was round-shaped and of shorter length, whereas LV- apoA-I-transduced ESC-derived cardiomyocytes were rod-shaped with long tapering ends and an extensive coverage of myofibrils, resembling the morphology of adult murine ventricular cardiomyocytes.(Figure 2C) [16], [17]. Real-time quantitative PCR consistently revealed that the expression of a panel of cardiac marker genes was significantly up-regulated in LV-apoA-I-transduced ESCs compared with LV-GFP-transduced ESCs, including α-MHC (4.3 fold, *p*<0.005), β-MHC (1.4 fold, *p*<0.05) and MLC2v (4.4 fold, *p*<0.005) (Figure 2D).

### apoA-I activates BMP4-SMAD1/5 signaling pathway in ESCs

**Figure 3:**
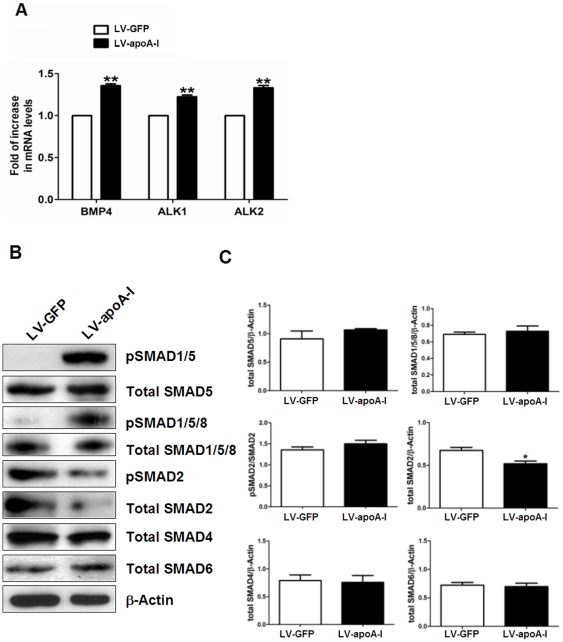
apoA-I gene transfer activates the BMP4-SMAD1/5 signaling cascade in undifferentiated ESCs. (A) The mRNA levels of bone morphogenic protein 4 (BMP4), activin receptor-like kinase 1 (ALK1) and activin receptor-like kinase 2 (ALK2) in the undifferentiated ESCs transduced with empty construct or apoA-I were evaluated by real-time quantitative PCR analysis using ribosomal protein S16 as internal control. (B) The phosphorylation status and total protein levels of the SMAD proteins were evaluated with Western blot analysis using β-actin as loading control. (C) The densitometry quantification of the Western blot results. Data shown as mean ± SEM from 3 independent experiments,* p<0.05; ** p<0.005.

In addition to its well recognized role in reverse cholesterol transport, HDL-C/apoA-I has been recently demonstrated to exert its effects on aortic endothelial cells by modulating expression of activin-like kinase receptors (ALK)1 and ALK2, thereby regulating BMP4 signaling [18]. It has been previously shown that BMP4 regulates cardiac differentiation of ESCs via the BMP/SMAD signaling pathway [19], [20], [21]. Therefore we also studied the ALK/BMP signaling pathway in apoA-I-enhanced cardiac differentiation of ESCs. Figure 3A shows that the mRNA levels of BMP4, ALK1 and ALK2 were significantly enhanced in LV-apoA-I-transduced ESCs compared with LV-GFP-transduced ESCs as determined by real-time quantitative PCR analysis. This suggests that apoA-I may activate BMP4 signaling at transcriptional levels. To corroborate and extend these observations, we performed Western blot analysis for SMAD signaling pathway: LV-apoA-I transduction in ESCs resulted in increased pSMAD1/5 signals, which was totally absent in LV-GFP-transduced ESCs (Figure 3B). To confirm this observation, another antibody against pSMAD1/5/8 was used. Similar result was observed except that trace amount of pSMAD1/5/8 could also be detected in the LV-GFP group (Figure 3B). Of note, the total SMAD2 protein level was significantly reduced in LV-apoA-I transduced ESCs compared with LV-GFP; there was nonetheless no statistically significant difference in pSMAD2/SMAD2 between these two groups (Figure 3B and C). In addition, no significant differences in levels of total SMAD4, SMAD5, SMAD1/5/8 and SMAD6 between LV-apoA-I and LV-GFP transduced ESCs were detected (Figure 3B and C).

### BMP4 inhibition abolished apoA-I stimulated cardiac differentiation of ESCs

**Figure 4:**
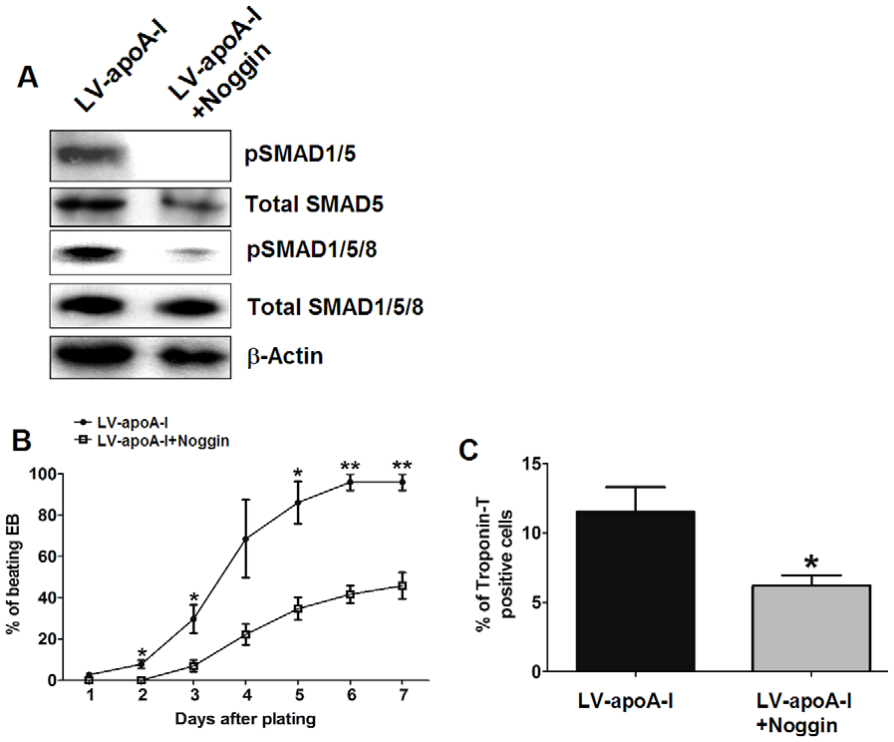
Inhibition of the BMP4 signaling pathway abolished the pro-cardiogenic effects of apoA-I gene transfer. The BMP4 signaling pathway was inhibited by the application of noggin (1 µ/ml) to the apoA-I- transduced ESCs upon plating. (A) The phosphorylation status of the differentiating cells was evaluated 24 hr after noggin treatment. (B) The appearance of beating clusters during the 8-day period of differentiation. (C) The percentage of troponin-T positive cells as determined by flow cytometry analysis. Data shown as mean ± SEM from 3 independent experiments, ^#^ p<0.05 comparing to the LV-apoA-I group; *p<0.05 and ** p<0.005 comparing to the LV-GFP group.

To confirm the involvement of a BMP4-SMAD signaling cascade in apoA-I-enhanced cardiac differentiation of ESCs, a BMP antagonist, noggin (1 ng/ml) that known to inhibit BMP-mediated cardiac differentiation [22], [23], [24], was added to differentiating LV-apoA-I-transduced ESCs. As shown in Figure 4, the application of noggin completely abrogated SMAD1/5 phosphorylation. Similar results were observed when another antibody against pSMAD1/5/8 was used despite trace amount of pSMAD1/5/8 could be observed in the LV-apoA-I+Noggin group (Figure 4A). On the other hand, there was no obvious change in the total protein amounts of SMAD5 and SMAD1/5/8 between the two groups (Figure 4A). In additions, noggin also abolished the LV-apoA-I enhanced cardiac differentiation as evidenced by a reduced percentage of embryoid bodies containing beating outgrowths (from 96±4% to 46±6%, *p*<0.005) and the number of troponin-T positive cells (from 12±2% to 5±2%, *p*<0.05) back to the control (LV-GFP) level (Figure 4B and C).

### Co-application of recombinant apoA-1 and BMP4 synergistically increases cardiac differentiation of ESCs

**Figure 5:**
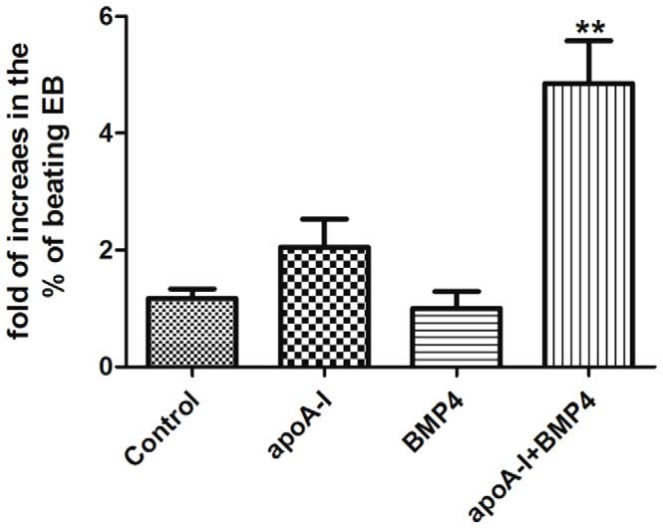
Synergistic effect of recombinant apoA-I and BMP4 on the cardiac differentiation of D3 ESCs. Untransduced D3 ESCs were subjected to cardiac differentiation in the presences or absence of recombinant apoA-1 (100 nM) and/or BMP4 (0.5 ng/ml). The appearance of beating clusters during the 8-day period of differentiation were recorded. Data shown as mean ± SEM from 3 independent experiments, ** p<0.005 comparing to the control group.

To demonstrate the synergetic effects of apoA-1 and BMP4 on promoting the cardiac differentiation of ESCs, untransduced D3 ESCs were differentiated in the presence or absence of either of both of the above factors. As showed in Figure 5, application of recombinant human apoA-1, but not BMP4 alone, only moderately increased the production of beating EBs, however, co-application of both factors significantly increased the presentage of beating EBs (∼5 fold, *p*<0.005). This clearly demonstrated that apoA-I promotes the cardiac differentiation via the BMP4 signally pathway.

### Maturation of calcium handling properties with apoA-I transduction

**Figure 6:**
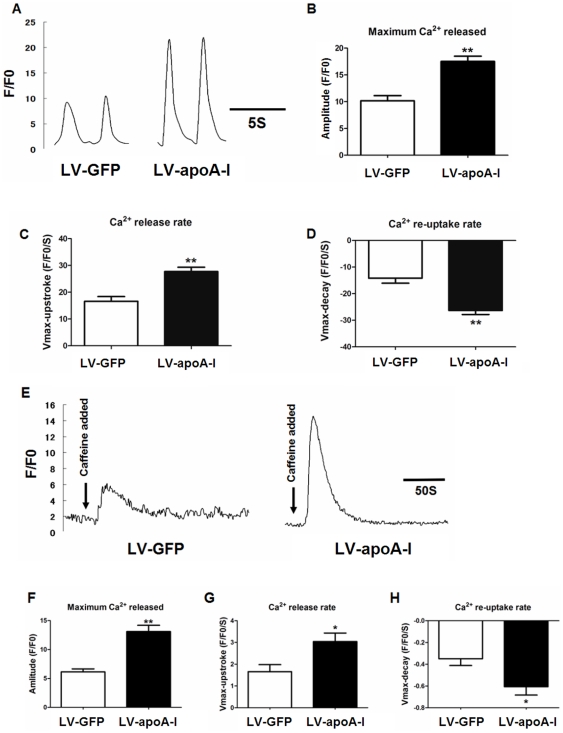
Calcium handling property of the cardiomyocytes derived from empty construct- and apoA-I-transduced ESCs. (A) Representative tracings of rhythmic spontaneous Ca2+ transients in cardiomyocytes derived from empty construct- and apoA-I- transduced cells. (B): Amplitude, (C) Maximal upstroke velocity (Vmax upstroke), (D) Maximal decay velocity (Vmax decay) of Ca2+ transients in the mESC-derived cardiomyocytes. (E) Representative tracings of caffeine-induced Ca2+ release from sarcoplasmic reticulum in cardiomyocytes derived from wild type, empty construct and apoA-I-1α transduced cells (right), demonstrating caffeine-sensitive Ca2+ stores and fractional release of total sarcoplasmic reticulum Ca2+ load during spontaneous activation. (F): Amplitude, (G) Maximal upstroke velocity (Vmax upstroke), (H) Maximal decay velocity (Vmax decay) of Ca2+ transients in the ESC-derived cardiomyocytes. Data shown as mean ± SEM from the recordings of 20–30 cells from 3–5 independent experiments, * p<0.05; ** p<0.005.

**Figure 7:**
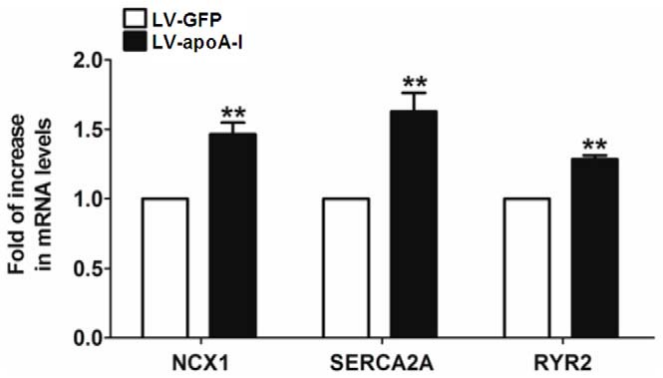
The expression of calcium handling components in cardiomyocytes. The mRNA levels of sodium/calcium exchanger (NCX1), sarcoplasmic reticulum Ca2+ ATPase (SERCA2A) and ryanodine receptor 2 (RYR2) of embryoid bodies (at d8 after plating) were evaluated by real-time quantitative PCR analysis using ribosomal protein S16 as internal control. Data shown as mean ± SEM from 3 independent experiments, ** p<0.005.

Spontaneous calcium oscillations in single cardiomyocytes were characterized with confocal microscope-based calcium transient recordings to determine whether apoA-I enhanced cardiac differentiation could influence the maturation of ESC-derived cardiomyocytes. While spontaneous calcium transients were observed in both LV- apoA-I- and LV-GFP-transduced ESC-derived cardiomyocytes, LV- apoA-I-transduced cells exhibited more mature calcium handling properties compared with their LV-GFP counterpart (Figure 6). Specifically, the amplitudes of calcium transient in LV-apoA-I-transduced ESC-derived cardiomyocytes, determined using a fluorescence intensity (F/F0) value measured against time, were 1.7 fold larger than those in LV-GFP-transduced counterpart (*p*<0.005)(Figure 6A and B). In kinetic analysis, LV-apoA-I-transduced ESC-derived cardiomyocytes had faster upstroke (LV-GFP: 16.6±1.8, n = 21 *vs.* LV-apoA-I: 27.7±1.6, n = 30, *p*<0.005) and decay velocities (LV-GFP: −14.2±1.9, n = 21 *vs.* LV-apoA-I: −26.3±1.5, n = 30, *p*<0.005) (Figure 6C and D). In addition brief exposure to caffeine (10 mM) that evaluated the calcium storage capacity of sarcoplasmic reticulum (SR) induced a robust surge in cytosolic calcium in both LV-apoA-I- and LV-GFP-transduced ESC-derived cardiomyocytes. This suggests the presence of a functioning internal calcium store (Figure 6E and F). The caffeine-induced calcium transient in LV-apoA-I-transduced ESC-derived cardiomyocytes nonetheless had a significantly larger amplitude (LV-GFP:6.1±0.5 *vs.* LV-apoA-I:13.1±1.1 F/F0, *p*<0.005)(Figure 6F), higher maximal upstroke velocity (LV-GFP 1.7±0.3 vs. LV-apoA-I:3.0±0.4 F/F0/s, *p*<0.05)(Figure 6G), and maximal decay velocity (LV-GFP: −0.3±0.1 vs. LV-apoA-I: −0.6±0.1 F/F0/s, *p*<0.05)(Figure 6H), compared with LV-GFP-transduced ESC-derived cardiomyocytes. This indicates a substantial increase in SR calcium content conferred by apoA-I transduction. To understand the mechanisms for these improvements in calcium handling, the expression of a panel of calcium handling proteins was determined using real-time quantitative PCR analysis (Figure 7). Consistent with the confocal calcium imaging result, LV-apoA-I-transduced ESCs had a higher expression of Na^+^-Ca^2+^ exchanger (NCX)-1 (1.4 fold, *p*<0.005), sarco/endoplasmic reticulum Ca^2+^-ATPase (SERCA)-2a (1.6 fold, *p*<0.005), and ryanodine receptor (RyR)-2 (1.3 fold, *p*<0.005) compared with LV-GFP-transduced ESCs (Figure 7).

### Exogenous apoA-I expression promotes cardiac differentiation of human iPSCs

**Figure 8:**
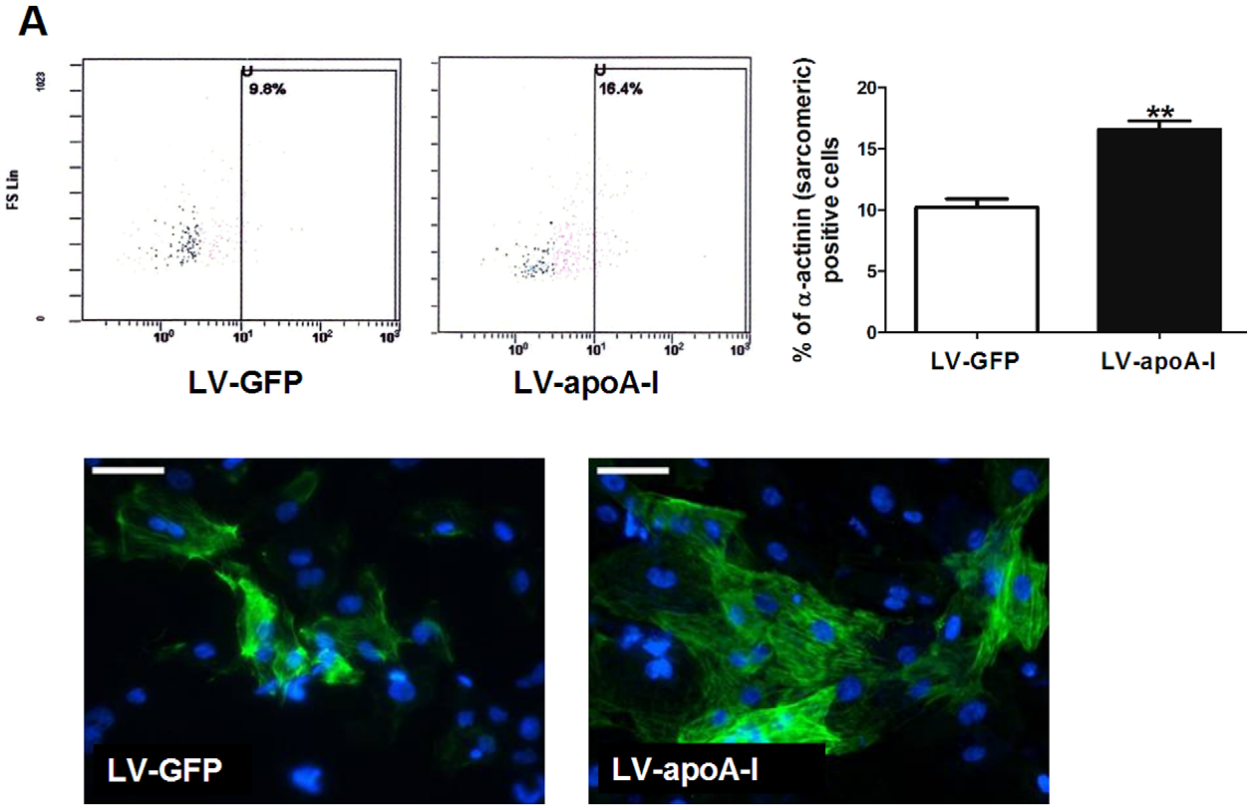
Effect of apoA-I gene transfer on the cardiac differentiation of IMR90 human IPSCs. (A) Percentage of ESC-derived cardiomyocytes (α-actinin (sarcomeric)-positive cells) 30 days after plating as determined by flow cytometry. (B) The beating EBs were digested into monolayers and the cardiomyocytes were identified with immunnohistochemistry using antibody specific to the cardiac troponin-T. Data shown as mean ± SEM from at least 3 independent experiments, *p<0.05; **p<0.005. Scale bar = 50 µm.

To determine whether enhanced expression of apoA-I exerts similar effects on other types of pluripotent stem cells, human iPSCs (line IMR90) transduced with LV-GFP or LV-apoA-I pseudoviral particles were subjected to cardiac differentiation. Although the transduction efficiency for IMR90 was much lower than that of D3 mouse ESCs (data not shown), LV-apoA-I transduction obviously increased the cardiac differentiation potential of IMR90. This was demonstrated by flow cytometery where LV-apoA-I transduction significantly increased the percentage of α- actinin (sarcomeric) positive cells from 10±1% to 16±1% (*p*<0.005) (Figure 8A). In addition, immunohistochemistry revealed that the numbers of cardiac troponin-T positive cells obviously increased in embryoid bodies derived from LV-apoA-I transduced groups (Figure 8B).

### Exogenouse apoA-I expression improves calcium homeostasis of human iPSC-derived cardiomyocytes

**Figure 9:**
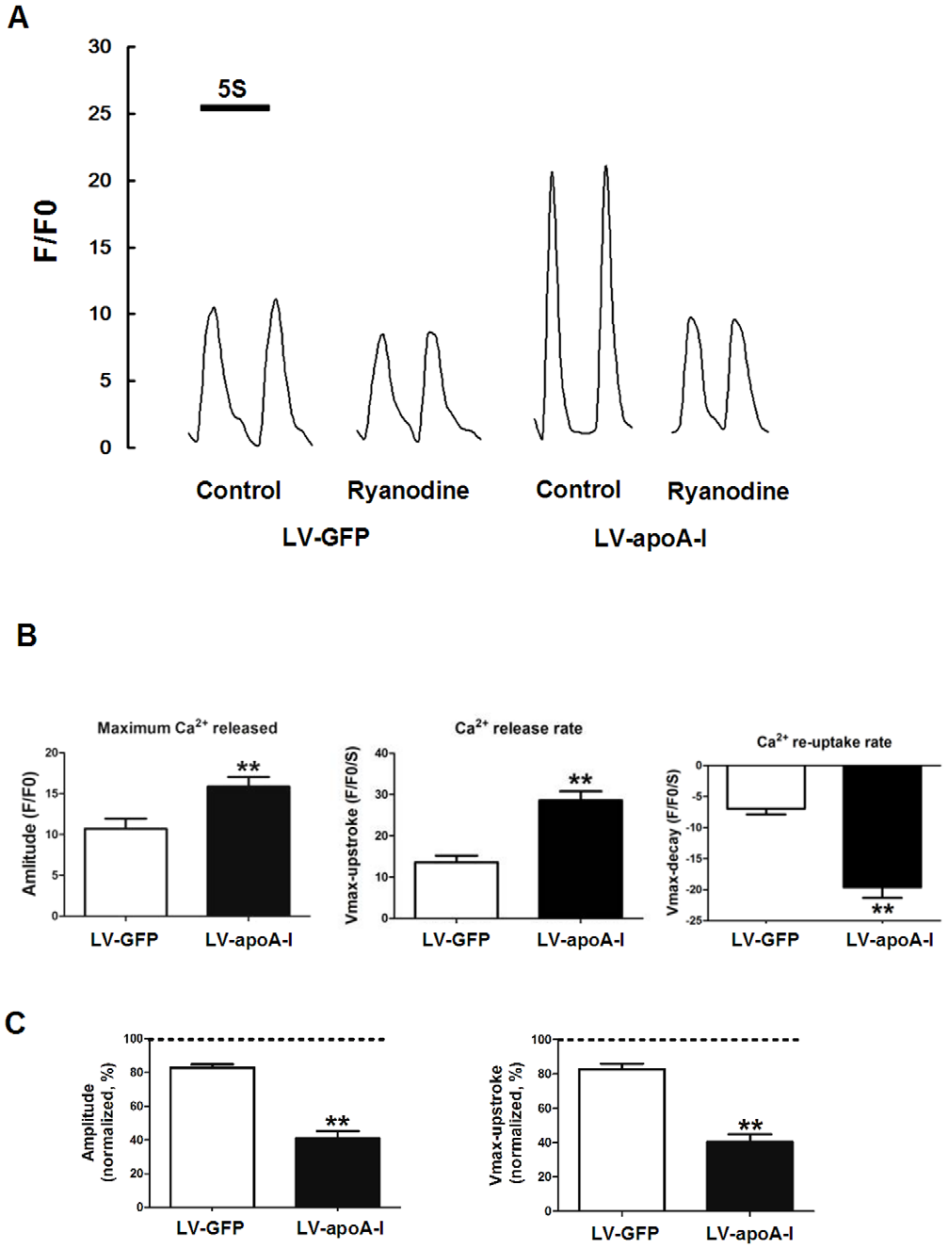
Calcium homeostasis in the cardiomyocytes derived from IMR90 (LV-GFP and LV-apoA-I). (A) representative tracings of the calcium transient in the absence (control) or presence of ryanodine. (B) statistical analysis of the amplitude, calcium release rate and calcium re-uptake rates of the IMR90-derived cardiomyocytes in the absence of Ryanodine. (C) amplitudes and calcium release rates after ryanodine application normalized to values recorded under control ryanodine-free conditions. Data shown as mean ± SEM from the recordings of 15 cells from 3–5 independent experiments, ** p<0.005.

The calcium handling properties of human iPSC-derived cardiomyocytes were also evaluated by confocal microscope. As shown in figure 9A and B, cardiomyocytes derived from LV-apoA-I transduced human iPSCs exhibited more mature calcium handling properties including a larger amplitude (LV-GFP:10.7±1.2 vs. LV-apoA-I:15.9±1.2 F/F0, *p*<0.005, n = 15), and higher maximal upstroke velocity (LV-GFP 13.6±1.6 vs. LV-apoA-I:28.6±2.2, F/F0/s, *p*<0.05, n = 15) and maximal decay velocity (LV-GFP: −7.0±0.9 vs. LV-apoA-I: −19.6±1.7, F/F0/s, *p*<0.05, n = 15), compared with LV-GFP-transduced ESC-derived cardiomyocytes. Unlike mESC-derived cardiomyocytes, human iPSC-derived cardiomyocytes (both LV-GFP and LV-apoA-I groups) were not responsive to caffeine. Nonetheless, application of 10 µM of ryanodine, a ryanodine receptor blocker, substantially reduced the amplitude (approximately 60% reduction compared with drug-free recordings, n = 15) and upstroke values (approximately 60% reduction compared with drug-free recordings, n = 15) of spontaneous calcium transients in the cardiomyocytes derived from the LV-apoA-I transduced group (Figure 9A and C). In contrast, cardiomyocytes derived from LV-GFP transduced human iPSCs exhibited only a modest response to ryanodine (<20% reductions in both amplitude and upstroke value of calcium transients) suggesting a more immature calcium handling apparatus.

## Discussion

To our knowledge, this is the first systematic study to evaluate the effects of apoA-I on cardiac differentiation of ESCs and human iPSCs. Our results demonstrate that exogenous expression of apoA-I promotes cardiac differentiation of pluripotent stem cells and enables maturation of calcium handling properties of pluripotent stem cell-derived cardiomyocytes. The main findings: (1) apoA-I over-expression promotes cardiac differentiation of mouse ESCs (in terms of the percentage of beating outgrowths, the number of cardiomyocytes as determined by flow cytometry), (2) apoA-I over-expression increases cardiac specific gene expression during ESC differentiation; (3) apoA-I-induced cardiac differentiation is dependent on a BMP4-SMAD1/5 signaling cascade; (4) apoA-I over-expression enhances the expression of calcium handling proteins (RyR2, NCX-1 and SERCA-2a) with a corresponding maturation of calcium handling properties in ESC-derived cardiomyocytes; (5) the cardiogenic effects of apoA-I are also observed in human iPSCs.

Accumulating evidence suggests that apoA-I plays a critical role not only in maintaining normal endothelial function [25], [26] but also in protecting cardiomyocytes from various types of insults including ischemic and re-perfusion injury [11], doxorubicin induced cardiomyocytes [10], and diabetic cardiomyopathy [12] through alternative mechanisms unrelated to cholesterol transportation. According to the current knowledge, the cardioprotective effects of apoA-1 and HDL-C appears to be largely contributed by its anti-inflammatory, anti-oxidative and anti-apoptotic action. In the present study, our data show that apoA-I is one of the upstream modulators of the BMP4 signaling pathway by concomitant up-regulation of expression of BMP4, and its receptors (ALK1 and ALK2), which in turn activates the SMAD1/5 signaling cascade. This apoA-I induced cardiac differentiation is mediated, at least in part, via this enhancement of BPM4 signaling, as evidenced by its complete abolition following application of noggin, an antagonist for BMP4, while co-application of recombinant apoA-1 and BMP4 synergistically promotes the cardiac differentiation of untransduced D3 ESCs. Similarly, Yao and co-workers have recently demonstrated in human aortic endothelial cells that HDL-C/apoA-I enhances BMP-signaling system by promoting expression of ALK2, a BMP-receptor, thus resulting in increased vascular endothelial growth factor (VEGF) and matrix Gla protein (MGP), essential for endothelial cell survival and prevention of vascular calcification, respectively [18]. This provides a mechanistic link between the HDL-C/apoA-I and BMP4 signaling pathway. As a member of the transforming growth factor (TGF)-β superfamily of proteins, BMPs play an important role in organogenesis of the cardiovascular system [22], [23] and tissue regeneration following injury [27]. Mechanistically, BMPs interacts with the membrane surface receptors, such as ALK1 and ALK2, and mediates the kinase activities of these receptors [27], [28]. This leads to the phosphorylation of receptor mediated-SMAD proteins, such as SMAD1, SMAD5 and SMAD8 [29], [30], [31], [32]. The phosporylated SMAD proteins then bind with SMAD4 to form the SMAD transcription factor complex which translocates into the nucleus and bind to the consensus DNA sequence to regulate the BMP target gene transcriptions [30], [32]. BMP4 and SMAD5 are critical to mesoderm formation and angiogenesis respectively.[27], [33], [34] BMPs has also been repeatedly shown to play an important role in cardiogenesis not only in embryonic stem cells, [19], [20], [21], [35], [36], [37], but also to promote c-kit positive bone marrow cells to adopt a cardiac fate [38], [39].

In adult cardiomyocytes, cyclic intracellular calcium transients underlying the excitation-contraction coupling are triggered by a process known as calcium-induced calcium release. During systole, a relative small calcium influx enters cells through L-type calcium channels and triggers a large calcium release from the internal calcium store, SR, through ryanodine receptors [40]. During the diastolic phase, cytosolic calcium is actively removed mainly through SERCA-2a back into SR and NCX-1 out of cells [41]. ESC-derived cardiomyocytes are nonetheless known to exhibit immature calcium dynamics including small cytosolic calcium transient amplitudes, slow rise and decay kinetics, and reduced calcium content of SR [3], [4], [41], [42], [43]. This is partly related to the underdeveloped SR and partly to the developmental expression profiles of calcium handling proteins in ESC-derived cardiomyocytes [3], [42]. In the present study, LV-apoA-I-transduced ESCs and iPSC-derived cardiomyocytes exhibited more mature calcium handling properties including larger calcium transients, and a faster rate of rise and decay of calcium transients. In addition, the LV-apoA-I-transduced ESC-derived cardiomyocytes appeared to have a larger internal store of calcium as evidenced by a larger amplitude of caffeine-mediated calcium release or ryanodine responsiveness. These improvements were associated with up-regulation of certain key calcium handling proteins (RyR2, NCX-1 and SERCA-2a). Mechanistically, the more mature calcium handling properties of LV-apoA-I-transduced mouse ESC-derived cardiomyocytes or human iPSC-derived cardiomyocytes may be due to accelerated cardiac differentiation or the direct effects of apoA-I on the cardiomyocytes and we are interesting in delineating between these two possibilities in the further study.

In summary, although the cardioprotective effects of apoA-I and HDL-C is well documented, little is known about its effects on the cardiac regeneration from pluripotent stem cells. By revealing role of apoA-I in promoting the cardiac differentiation of ESCs and iPSCs and the involvement of BMP4 signaling cascade, our study provided new insights for the therapeutic values of apoA-I gene transfer in regenerative medicine. First, the human iPSCs-derived cardiomyocytes that stably expressing the apoA-I is expected to be a functionally improved cell sources for transplantation, more importantly, after transplantation, these cells may act as an internal sources of the apoA-I that may lead to the increased local HDL-C levels in the infarct sites that could be beneficial to the recovery of myocardial infraction.

## Materials and Methods

### Ethics Statement

The present study was conducted entirely on commercially available cell-lines, no human participants or animals were involved, as such, ethics approval was not required for the present study.

### Murine ESC culture and cardiac differentiation

A murine ESC-line D3 (CRL-1934, American Type Culture Collection, Manassas, VA) was used in this study and cultured on irradiation-inactivated mouse embryonic feeders as previously described [2], [4], [44]. To induce cardiac differentiation, embryoid bodies (∼800 ESCs in 20 µL culture medium in the absence of leukemia inhibitory factors) were formed using a hanging drop method followed by 7 days in suspension culture. After the hanging drop stage, the embryoid bodies were then plated onto 0.1% gelatin-coated tissue culture dishes [2], [4], [44]. Whenever necessary, recombinant human apoA-I (100 nM) (Sigma-Aldrich, St. Louis, MO), bone morphogenetic protein-4 (BMP4) (0.5 ng/ml) or noggin (1 µg/ml), an inhibitor of the bone morphogenetic protein (BMP)-pathway, was supplemented during differentiation.

### Human iPSC culture and cardiac differentiation

Undifferentiated human iPSCs (IMR90, WiCell Research Institute, Madison, Wisconsin, USA) were maintained in a serum-free and feeder-free culture system (StemPro hESC SFM system) (Invitrogen Gibco) according to the manufacture's instructions. When 80% confluence was reached, cells were treated with dispase (1 mg/ml) for 5 minutes at 37°C followed by mechanical scraping. Cell aggregates were allowed to form embryoid bodies in suspension culture with StemPro hESC medium for 5 days. Embryoid bodies were then plated onto gelatin-coated plates in a basal medium (StemPro34, Invitrogen) supplemented with 2 mM glutamine, 4 mM monothioglycerol, 50 µg/ml ascorbic acid, and 0.5 ng/ml BMP4 according to a previous protocol [21].

### Lentivirus-mediated gene transfer

To exogenously express apoA-I in murine ESCs and human iPSCs, full-length cDNA of human apoA-I obtained from Origene (SC 110828, Origene technologies, Rockville, MD) was subcloned into the lentiviral vector pHRST-CMV-IRES-GFP (pLV-GFP) with blunt-end-ligation to generate pHRST-CMV-apoA-I-IRES-GFP (pLV-apoA-I). The constitutively active CMV promoter enables transgene expression in all cell types in both undifferentiated and differentiated states. The internal ribosomal entry site (IRES) allows the simultaneous translation of two transgenes with a single transcript and, in our experiments, green fluorescence protein and an apoA-I construct. The production of lentiviruses has been described previously [44]. Briefly, the lentiviral vector constructs (pLV-apoA-I and pLV-GFP (empty cassette)) were co-transferred with the virus-packaging plasmid (pCMV-dR8.91) and envelope plasmid (pMD2G) into HEK293FT cells (Invitrogen) with lipofectamine 2000 (Invitrogen) mediated transfection. The supernatant was collected 48 hours after transfection and concentrated by high-speed centrifugation (20,000 g for 2 hours at 4°C). For transduction, undifferentiated mouse ESCs and human iPSCs were incubated with 50 ul supernatant without feeder cells for 12 hours.

### Flow cytometry analysis

The percentage of ESC-derived cardiomyocytes was quantified using flow cytometry analysis on day 7 after plating for mouse ESCs and on day 30 for human iPSCs. Briefly, embryoid bodies were dissociated to single cells with collagenase B (1 mg/ml). Cells were fixed and permeabilized using a Cytofix/Cytoperm permeabilization kit (BD Biosciences, San Diego, CA), and stained with monoclonal anti-troponin T antibody (dilution 1∶100; NeoMarker, Fremont, CA) or monoclonal anti-α-actinin (sarcomeric) antibody (A7811,dilution 1∶100; Sigma) followed by a secondary antibody, anti-mouse IgG H+L-PE (dilution 1∶100; Beckman Coulter, Fullerton, CA, USA). Analysis was performed using a Beckman Coulter FC500 flow cytometer. The primary antibody, IgG_1_, was used as an isotypic control to determine background signal.

### Real-time quantitative polymerase chain reaction analysis

Total RNA was isolated and reverse transcribed using an Ilustra RNAspin Mini Kit (GE healthcare). Reverse transcription (RT) was performed with 0.5 µg of total RNA using a QuantiTect® reverse transcription kit (Qiagen, Hilden, Germany) according to the manufacturer's instructions. Oligonucleotides of the genes of interest in quantitative polymerase chain reaction (PCR) are listed in Table 1. Quantitative PCR analysis was performed with DNA engine opticon 2 real-time PCR detection system (Bio-Rad, Hercules, USA) using iQ SYBR Green Supermix (Bio-Rad, Hercules, USA). The relative quantification of the PCR products was performed according to the 2^−ΔΔCt^ method, using mouse ribosomal protein S16 (S16) as an internal control, where ΔΔCt = [(Ct_target gene_−Ct_S16_)_LV-apoA-I_−(Ct _target gene_−Ct _S16_)_LV-GFP_].

**Table 1:** Primers used in the real-time quantitative RT-PCR analysis.

Genes of interest	Sequence 5′→3′	
BMP4	Forward	TCGTTACCTCAAGGGAGTGG
	Reverse	ATGCTTGGGACTACGTTTGG
ALK1	Forward	GTCAAGAAGCCTCCAGCAAC
	Reverse	CCTTGTCTCTTGCCTGAACC
ALK2	Forward	GATACGGTTAGCTGCCTTCG
	Reverse	TGCAGCACTGTCCATTCTTC
MHC-A	Forward	GATGCCCAGATGGCTGACTT
	Reverse	GGTCAGCATGGCCATGTCCT
MHC-B	Forward	GCCAACACCAACCTGTCCAAGTTC
	Reverse	TGCAAAGGCTCCAGGTCTGAGGGC
MLC2V	Forward	TTCTCAACGCATTCAAGGTG
	Reverse	CTGGTCGATCTCCTCTTTGG
GATA4	Forward	TCTCCCAGGAACATCAAAACC
	Reverse	GTGTGAAGGGGTGAAAAGG
Nkx2.5	Forward	GCTACAAGTGCAAGCGACAG
	Reverse	GGGTAGGCGTTGTAGCCATA
NCX1	Forward	TGTGTTTACGTGGTCCCTGA
	Reverse	CTCCACAACTCCAGGAGAGC
SERCA2A	Forward	GATCACACCGCTGAATCTG
	Reverse	AGTATTGCGGGTTGTTCCAG
RYR2	Forward	TCTTAGCCATCCTCCACACC
	Reverse	TCCGTCAAACTCCAACTTCC
S16	Forward	CATCTCAAAGGCCCTGGTAG
	Reverse	CCAAACTTTTTGGATTCGCA

Abbreviation: ALK1: ; ALK2: ; α-MHC: alpha-myosin heavy chain; β-MHC: beta-myosin heavy chain; MLC2v: myosin light chain 2v; GATA4: GATA binding protein 4; Nkx2.5: NK2 transcription factor related, locus 5; NCX: sodium calcium exchanger; SERCA-2a: sarcoplasmic reticulum Calcium ATPase 2a; RyR2: ryanodine receptor 2.

### Western blot analysis

Total proteins were extracted from undifferentiated ESCs and/or embryoid bodies using a lysis buffer containing 1% Triton X-100, 1% sodium deoxycolate, 0.1%SDS, 150 mM sodium chloride, and 10 mM sodium phosphate, at pH 7.2. Protein (20 µg) was loaded in a 10% SDS page, and electrically transferred onto a PVDF membrane that was probed with the antibodies listed in Table 2. Detection was performed with a standard ECL based system.

**Table 2:** Primary Antibodies used for Western Blot analysis.

Antibody (origin)	Catalog number	Vendor
Anti-APOA1(mouse monoclonal)	SC-58230	Santa CruzBiotechnology
HRP conjugated-anti-β-Actin (mouse monoclonal)	SC-47778 HRP	Santa CruzBiotechnology
Anti-Phospho-SMAD1/5 (Ser463/465)(Rabbit monoclonal)	# 9516	Cell Signaling Technology
Anti-Phospho-SMAD1 (Ser463/465)/SMAD5 (Ser463/465)/SMAD8 (Ser426/428)(Rabbit polyclonal)	#9511	Cell Signaling Technology
Anti-Phospho-SMAD2 (Ser465/467)(Rabbit monoclonal)	# 3108	Cell Signaling Technology
Anti-SMAD2(Rabbit monoclonal)	# 3122	Cell Signaling Technology
Anti-SMAD5(Rabbit polyclonal)	# 9517	Cell Signaling Technology
Anti-SMAD1/5/8(Rabbit polyclonal)	sc-6031-R	Santa CruzBiotechnology
Anti-SMAD4 Antibody(Rabbit polyclonal)	# 9515	Cell Signaling Technology
Anti-SMAD6 Antibody(Rabbit polyclonal)	# 9519	Cell Signaling Technology

### Immunofluorescence staining

Beating embryoid bodies from mouse ESCs and human iPSCs were dissociated, plated onto glass coverslips, and fixed with 2% paraformaldehyde for 48 hours at 4°C. After fixation, cells were washed twice with PBS and incubated in a blocking buffer (2% BSA, PBS, 0.1% Triton-X100) for 2 hours. Cells were then incubated with murine monoclonal anti-troponin T (dilution 1∶100; NeoMarker, Fremont, CA) at 4°C overnight. After removal of unbound antibodies with washing buffer, cells were incubated with Alexa-488 conjugated anti-rabbit secondary antibodies (Invitrogen) (1∶100 dilution in washing buffer) for 1 hour at room temperature. Cells were then mounted on glass slides with a mounting medium containing DAPI. Presence of the immune-complex was examined using fluorescent microscopy.

### Confocal Calcium Imaging

Beating outgrowths from D3 murine ESC-derived embryoid bodies (day 8) and human iPSC- derived embryoid bodies (day 30) were micro-surgically dissected using a glass knife, followed by incubation in collagenase B (1 mg/ml)(Roche) at 37°C for 30 minutes with occasional dispersion by pipetting up and down. Single cardiomyocytes were then plated on glass coverslips for confocal calcium imaging [2], [4], [44], [45]. To assess calcium-handling properties, ESC-derived cardiomyocytes were loaded with 5 µM Rhod-2 AM (Invitrogen) for 45 min at 37°C in Tyrode solution containing 140 mM NaCl, 5 mM KCl, 1 mM MgCl_2_, 1.8 mM CaCl_2_, 10 mM glucose and 10 mM HEPES at pH 7.4. Calcium transients of single ESC-derived cardiomyocytes were recorded using a confocal imaging system (Olympus Fluoview System version 4.2 FV300 TIEMPO) mounted on an upright Olympus microscope (IX71) with temporal resolution of the line scan at 274 frames per second. Signals were then quantified as the background-subtracted fluorescence intensity changes normalized to the background-subtracted baseline fluorescence using Image J software.

### Statistical Analysis

Continuous variables are expressed as mean ± SEM. Statistical comparisons were performed using Student's *t*-test. Calculations were performed with GraphPad Prism 5 (GraphPad, La Jolla, CA). A *P*-value <0.05 was considered statistically significant.

## References

[1] Siu CW, Moore JC, Li RA (2007). Human embryonic stem cell-derived cardiomyocytes for heart therapies.. Cardiovasc Hematol Disord Drug Targets.

[2] Au KW, Liao SY, Lee YK, Lai WH, Ng KM (2009). Effects of iron oxide nanoparticles on cardiac differentiation of embryonic stem cells.. Biochem Biophys Res Commun.

[3] Lieu DK, Liu J, Siu CW, McNerney GP, Tse HF (2009). Absence of transverse tubules contributes to non-uniform Ca(2+) wavefronts in mouse and human embryonic stem cell-derived cardiomyocytes.. Stem Cells Dev.

[4] Lee YK, Ng KM, Chan YC, Lai WH, Au KW Triiodothyronine promotes cardiac differentiation and maturation of embryonic stem cells via the classical genomic pathway.. Mol Endocrinol.

[5] Liao SY, Liu Y, Siu CW, Zhang Y, Lai WH Pro-arrhythmic Risk of Embryonic Stem Cell-Derived Cardiomyocytes Transplantation in Infarcted Myocardium.. Heart Rhythm.

[6] Gordon DJ, Rifkind BM (1989). High-density lipoprotein–the clinical implications of recent studies.. N Engl J Med.

[7] Lane DM (2001). Third report of the National Cholesterol Education Program (NCEP. III): A switch from dietary modification to risk factor assessment.. Curr Opin Investig Drugs.

[8] Mineo C, Deguchi H, Griffin JH, Shaul PW (2006). Endothelial and antithrombotic actions of HDL.. Circ Res.

[9] Mineo C, Shaul PW (2003). HDL stimulation of endothelial nitric oxide synthase: a novel mechanism of HDL action.. Trends Cardiovasc Med.

[10] Frias MA, Lang U, Gerber-Wicht C, James RW Native and reconstituted HDL protect cardiomyocytes from doxorubicin-induced apoptosis.. Cardiovasc Res.

[11] Theilmeier G, Schmidt C, Herrmann J, Keul P, Schafers M (2006). High-density lipoproteins and their constituent, sphingosine-1-phosphate, directly protect the heart against ischemia/reperfusion injury in vivo via the S1P3 lysophospholipid receptor.. Circulation.

[12] Van Linthout S, Spillmann F, Riad A, Trimpert C, Lievens J (2008). Human apolipoprotein A-I gene transfer reduces the development of experimental diabetic cardiomyopathy.. Circulation.

[13] Baroukh N, Lopez CE, Saleh MC, Recalde D, Vergnes L (2004). Expression and secretion of human apolipoprotein A-I in the heart.. FEBS Lett.

[14] Zannis VI, Cole FS, Jackson CL, Kurnit DM, Karathanasis SK (1985). Distribution of apolipoprotein A-I, C-II, C-III, and E mRNA in fetal human tissues. Time-dependent induction of apolipoprotein E mRNA by cultures of human monocyte-macrophages.. Biochemistry.

[15] Moore JC, van Laake LW, Braam SR, Xue T, Tsang SY (2005). Human embryonic stem cells: genetic manipulation on the way to cardiac cell therapies.. Reprod Toxicol.

[16] Maltsev VA, Wobus AM, Rohwedel J, Bader M, Hescheler J (1994). Cardiomyocytes differentiated in vitro from embryonic stem cells developmentally express cardiac-specific genes and ionic currents.. Circ Res.

[17] Maltsev VA, Wolff B, Hess J, Werner G (1994). Calcium signalling in individual T-cells measured by confocal microscopy.. Immunol Lett.

[18] Yao Y, Shao ES, Jumabay M, Shahbazian A, Ji S (2008). High-density lipoproteins affect endothelial BMP-signaling by modulating expression of the activin-like kinase receptor 1 and 2.. Arterioscler Thromb Vasc Biol.

[19] Taha MF, Valojerdi MR (2008). Effect of bone morphogenetic protein-4 on cardiac differentiation from mouse embryonic stem cells in serum-free and low-serum media.. Int J Cardiol.

[20] Takei S, Ichikawa H, Johkura K, Mogi A, No H (2009). Bone morphogenetic protein-4 promotes induction of cardiomyocytes from human embryonic stem cells in serum-based embryoid body development.. Am J Physiol Heart Circ Physiol.

[21] Yang L, Soonpaa MH, Adler ED, Roepke TK, Kattman SJ (2008). Human cardiovascular progenitor cells develop from a KDR+ embryonic-stem-cell-derived population.. Nature.

[22] Nakajima Y, Yamagishi T, Ando K, Nakamura H (2002). Significance of bone morphogenetic protein-4 function in the initial myofibrillogenesis of chick cardiogenesis.. Dev Biol.

[23] Jamali M, Karamboulas C, Rogerson PJ, Skerjanc IS (2001). BMP signaling regulates Nkx2-5 activity during cardiomyogenesis.. FEBS Lett.

[24] Gianakopoulos PJ, Skerjanc IS (2009). Cross talk between hedgehog and bone morphogenetic proteins occurs during cardiomyogenesis in P19 cells.. In Vitro Cell Dev Biol Anim.

[25] Barter PJ, Nicholls S, Rye KA, Anantharamaiah GM, Navab M (2004). Antiinflammatory properties of HDL.. Circ Res.

[26] Tao R, Hoover HE, Honbo N, Kalinowski M, Alano CC High-density lipoprotein determines adult mouse cardiomyocyte fate after hypoxia-reoxygenation through lipoprotein-associated sphingosine 1-phosphate.. Am J Physiol Heart Circ Physiol.

[27] Sieber C, Kopf J, Hiepen C, Knaus P (2009). Recent advances in BMP receptor signaling.. Cytokine Growth Factor Rev.

[28] Chen D, Zhao M, Mundy GR (2004). Bone morphogenetic proteins.. Growth Factors.

[29] Miyazono K, Kamiya Y, Morikawa M (2010). Bone morphogenetic protein receptors and signal transduction.. J Biochem.

[30] Schmierer B, Hill CS (2007). TGFbeta-SMAD signal transduction: molecular specificity and functional flexibility.. Nat Rev Mol Cell Biol.

[31] Heldin CH, Miyazono K, ten Dijke P (1997). TGF-beta signalling from cell membrane to nucleus through SMAD proteins.. Nature.

[32] Massague J, Seoane J, Wotton D (2005). Smad transcription factors.. Genes Dev.

[33] Winnier G, Blessing M, Labosky PA, Hogan BL (1995). Bone morphogenetic protein-4 is required for mesoderm formation and patterning in the mouse.. Genes Dev.

[34] Yang X, Castilla LH, Xu X, Li C, Gotay J (1999). Angiogenesis defects and mesenchymal apoptosis in mice lacking SMAD5.. Development.

[35] Kado M, Lee JK, Hidaka K, Miwa K, Murohara T (2008). Paracrine factors of vascular endothelial cells facilitate cardiomyocyte differentiation of mouse embryonic stem cells.. Biochem Biophys Res Commun.

[36] Taha MF, Valojerdi MR, Mowla SJ (2007). Effect of bone morphogenetic protein-4 (BMP-4) on cardiomyocyte differentiation from mouse embryonic stem cell.. Int J Cardiol.

[37] Wu J, Kubota J, Hirayama J, Nagai Y, Nishina S (2010). p38 Mitogen-activated protein kinase controls a switch between cardiomyocyte and neuronal commitment of murine embryonic stem cells by activating myocyte enhancer factor 2C-dependent bone morphogenetic protein 2 transcription.. Stem Cells Dev.

[38] Degeorge BR, Rosenberg M, Eckstein V, Gao E, Herzog N (2008). BMP-2 and FGF-2 synergistically facilitate adoption of a cardiac phenotype in somatic bone marrow c-kit+/Sca-1+ stem cells.. Clin Transl Sci.

[39] Lagostena L, Avitabile D, De Falco E, Orlandi A, Grassi F (2005). Electrophysiological properties of mouse bone marrow c-kit+ cells co-cultured onto neonatal cardiac myocytes.. Cardiovasc Res.

[40] Bers DM (2006). Cardiac ryanodine receptor phosphorylation: target sites and functional consequences.. Biochem J.

[41] Satin J, Itzhaki I, Rapoport S, Schroder EA, Izu L (2008). Calcium handling in human embryonic stem cell-derived cardiomyocytes.. Stem Cells.

[42] Liu J, Fu JD, Siu CW, Li RA (2007). Functional sarcoplasmic reticulum for calcium handling of human embryonic stem cell-derived cardiomyocytes: insights for driven maturation.. Stem Cells.

[43] Liu J, Lieu DK, Siu CW, Fu JD, Tse HF (2009). Facilitated maturation of Ca2+ handling properties of human embryonic stem cell-derived cardiomyocytes by calsequestrin expression.. Am J Physiol Cell Physiol.

[44] Ng KM, Lee YK, Chan YC, Lai WH, Fung ML Exogenous expression of HIF-1alpha promotes cardiac differentiation of embryonic stem cells.. J Mol Cell Cardiol.

[45] Yeh BK, Hoffman BF (1968). The Ionic Basis of Electrical Activity in Embryonic Cardiac Muscle.. J Gen Physiol.

